# Spontaneous Recovery from Unresponsive Wakefulness Syndrome to a Minimally Conscious State: Early Structural Changes Revealed by 7-T Magnetic Resonance Imaging

**DOI:** 10.3389/fneur.2017.00741

**Published:** 2018-01-17

**Authors:** Xufei Tan, Jian Gao, Zhen Zhou, Ruili Wei, Ting Gong, Yuqin Wu, Kehong Liu, Fangping He, Junyang Wang, Jingqi Li, Xiaotong Zhang, Gang Pan, Benyan Luo

**Affiliations:** ^1^Department of Neurology and Brain Medical Centre, The First Affiliated Hospital, School of Medicine, Zhejiang University, Hangzhou, China; ^2^Department of Rehabilitation, Hangzhou Hospital of Zhejiang CAPR, Hangzhou, China; ^3^College of Computer Science and Technology, Zhejiang University, Hangzhou, China; ^4^Center for Brain Imaging Science and Techonology, College of Biomedical Engineering and Instrumental Science, Zhejiang University, Hangzhou, China; ^5^Interdisciplinary Institute of Neuroscience and Technology, Qiushi Academy for Advanced Studies, Zhejiang University, Hangzhou, China; ^6^School of Medicine, Zhejiang University, and Collaborative Innovation Center for Brain Science, Hangzhou, China

**Keywords:** disorders of consciousness, recovery, temporoparietal junction, cuneus, ultra-high field (7 T), structural changes

## Abstract

**Background:**

Determining the early changes of brain structure that occur from vegetative state/unresponsive wakefulness syndrome (VS/UWS) to a minimally conscious state (MCS) is important for developing our understanding of the processes underlying disorders of consciousness (DOC), particularly during spontaneous recovery from severe brain damage.

**Objective:**

This study used a multi-modal neuroimaging approach to investigate early structural changes during spontaneous recovery from VS/UWS to MCS.

**Methods:**

The Coma Recovery Scale-Revised (CRS-R) score, 24-h electroencephalography (EEG), and ultra-high field 7-T magnetic resonance imaging were used to investigate a male patient with severe brain injury when he was in VS/UWS compared to MCS. Using white matter connectometry analysis, fibers in MCS were compared with the same fibers in VS/UWS. Whole-brain analysis was used to compare all fibers showing a 10% increase in density with each other as a population.

**Results:**

Based on connectometry analysis, the number of fibers with increased density, and the magnitude of increase in MCS compared to VS/UWS, was greatest in the area of the temporoparietal junction (TPJ), and was mostly located in the right hemisphere. These results are in accordance with the active areas observed on 24-h EEG recordings. Moreover, analysis of different fibers across the brain, showing at least a 10% increase in density, revealed that altered white matter connections with higher discriminative weights were located within or across visual-related areas, including the cuneus_R, calcarine_R, occipital_sup_R, and occipital_mid_R. Furthermore, the temporal_mid_R, which is related to the auditory cortex, showed the highest increase in connectivity to other areas. This was consistent with improvements in the visual and auditory components of the CRS-R, which were greater than other improvements.

**Conclusion:**

These results provide evidence to support the important roles for the TPJ and the visual and auditory sensory systems in the early recovery of a patient with severe brain injury. Our findings may facilitate a much deeper understanding of the mechanisms underlying conscious-related processes and enlighten treatment strategies for patients with DOC.

## Introduction

Understanding the changes in brain connectivity networks associated with severe brain damage, such as vegetative state/unresponsive wakefulness syndrome (VS/UWS) (1) and minimally conscious state (MCS) (2), is critical to the study of disorders of consciousness (DOC) (3). Changes occurring during the spontaneous recovery of brain function are particularly informative (4). Therefore, DOC patients provide us with a unique opportunity to investigate aspects of brain network connectivity that are directly related to consciousness (5).

Emphasis on white matter is founded in the so-called “disconnection hypothesis,” which posits that lesions in the white matter microstructure lead to interruption of communication between regions of the cortex, thus resulting in poorer cognitive performance (6, 7). Thus, white matter destruction is an important determinant of cognitive impairment after brain injury, although conventional neuroimaging methods tend to underestimate its true extent (8). In recent years, *in vivo* diffusion tensor imaging has been increasingly used to characterize the brain white matter at greater resolution in patients with DOC (9, 10). However, DOC is better described as a clinical phenomenon than a disease; the features of DOC vary widely between different individuals. To understand changes in brain networks during recovery from DOC, it is important to study a single patient over time, as evidenced by variability in the major imaging correlates of recovery in longitudinal studies of patients with clinical impairment.

Recently, a new method, referred to as connectometry, was developed, which improves fiber tracking by more accurately reflecting the structure and density of the white matter tracts, while also accounting for crossing fibers and partial volume effects (11, 12). In the present study, we aimed to use connectometry to elucidate early changes in the white matter tract during spontaneous recovery from VS/UWS to MCS in a patient with trauma. Ultra-high field (7 T) magnetic resonance imaging (MRI) was utilized to gain higher resolution, signal-to-noise ratio, and contrast-to-noise ratio (13). Resultant data should provide us with a better understanding of the recovery of consciousness and help promote an early prognosis of recovery outcome in patients with DOC. It will further provide supporting evidence to build a close-loop cyborg intelligent system for rehabilitation (42, 43).

## Background

This study involved a 23-year-old male patient, who was a factory worker with no known confounding neurological condition. He had suffered head trauma in a traffic accident one and a half months prior to the start of the study. He was found unconscious and behaviorally unresponsive and was urgently delivered to the local hospital. At that time, a computed tomography showed a hematoma involving the left basal ganglia, subarachnoid hemorrhage, and ventricular hemorrhage. Furthermore, there were multiple bone fractures over the entire body. One and a half months after the initial trauma, the patient exhibited impaired consciousness, with a Coma Recovery Scale-Revised (CRS-R) score of 4 (auditory function: 0, visual function: 0, motor function: 2, verbal function: 0, communication: 0, and arousal: 2). No obvious sleep spindle wave was found on 24-h dynamic electroencephalography (EEG). At that time, the first MRI scan was performed under sedation at 7.0 T. After a series of symptomatic and supportive treatments, such as using appropriate antibiotics to control infection, sufficient enteral feeding to maintain adequate nutrition and passive exercises to prevent venous thrombosis, the patient gradually recovered consciousness without surgery. Five months after onset, the patient’s CRS-R score had recovered to 16 (4/4/4/0/1/3) and 24-h dynamic EEG showed obvious sleep stages and spindle waves. A second ultra-high field MRI scan was then carried out. Our protocol was approved by the Ethics Committee of the First Affiliated Hospital, School of Medicine, Zhejiang University, and written informed consent was obtained from the legal guardian of the patient to allow the patient to participate in the study and for this case report to be published.

## Imaging Protocols

Imagine was performed on a 7 T Siemens Magnetom scanner (Siemens Healthcare, Erlangen, Germany), equipped with an SC72 body gradient (70 mT/m gradient strength and 200 T/m/s slew rate) and a Nova 1Tx/32Rx head coil (Nova Medical, USA). Whole-brain T1-weighted magnetization prepared rapid acquisition gradient echo (MPRAGE) images were acquired with a 0.75 mm isotropic resolution: repetition time (TR) = 2,590 ms; echo time (TE) = 2.48 ms, flip angle = 7°, field of view (FOV) = 198 ×198, matrix = 264 × 264, bandwidth = 250 Hz/pixel, GRAPPA factor = 2, acquisition time: 5′30′′. Simultaneous multiple slice (SMS) diffusion images were also acquired with 1.25 mm isotropic resolution, covering the entire brain: multi-band factor of 2, GRAPPA factor = 3, 112 slices, unipolar diffusion weighting gradients, TE = 66.2 ms, TR = 5,100 ms, phase encoding direction: anterior–posterior and posterior–anterior, respectively, bandwidth = 1,566 Hz/pixel, 30 diffusion directions (*b*-value = 1,000 s/mm^2^), 3 interspersed b0 images (non-diffusion weighted, *b*-value = 0 s/mm^2^), and the total acquisition time of 3′34′′.

## Data Processing

Diffusion preprocessing for motion, susceptibility, and eddy current distortion corrections were performed with FSL’s eddy and topup tools (14). Diffusion post-processing and analysis were conducted using DSI Studio (http://dsi-studio.labsolver.org). Spin distribution functions were reconstructed in Montreal Neurological Institute (MNI) space using q-space diffeomorphic reconstruction (15). A diffusion sampling length ratio of 1.25 was used, and the output resolution was 2 mm. We further explored differences between the patient’s first and the second scans by performing connectometry (16). Local fiber bundles with proportional reductions greater than 5, 10, 15, and 20%, and proportional increases greater than 10, 20, 30, and 40% were connected using a deterministic fiber-tracking algorithm (17). Tracts with a connected length greater than 30 mm were included.

As this connectometry method only provided us with information relating to an increase or decrease of fiber intensity relative to other fibers, we later subdivided the white matter in the patient’s brain according to the automated anatomical labeling (AAL)-90 atlas to determine differences among different fibers. In order to analyze as many fibers as possible, we selected those showing an increase of at least 10% for subsequent analyses. We selected each region of interest (ROI) of the AAL-90 atlas as a seed region and traced the fibers using a deterministic fiber-tracking algorithm (17). The angular threshold was 45°, step size was 0.5 mm and the anisotropy threshold was 0.05. A total of 100,000 seeds were placed. This process was then repeated for all other ROIs. The AAL-90 was used for brain parcelation, and the connectivity matrix was calculated by using the count of all connecting tracks. Connectivity matrix generation and graph theoretical analysis were conducted using DSI Studio. The matrix was then analyzed with Circos (18).

## Results

### Microstructural Abnormalities in the Patient

**Figure 1:**
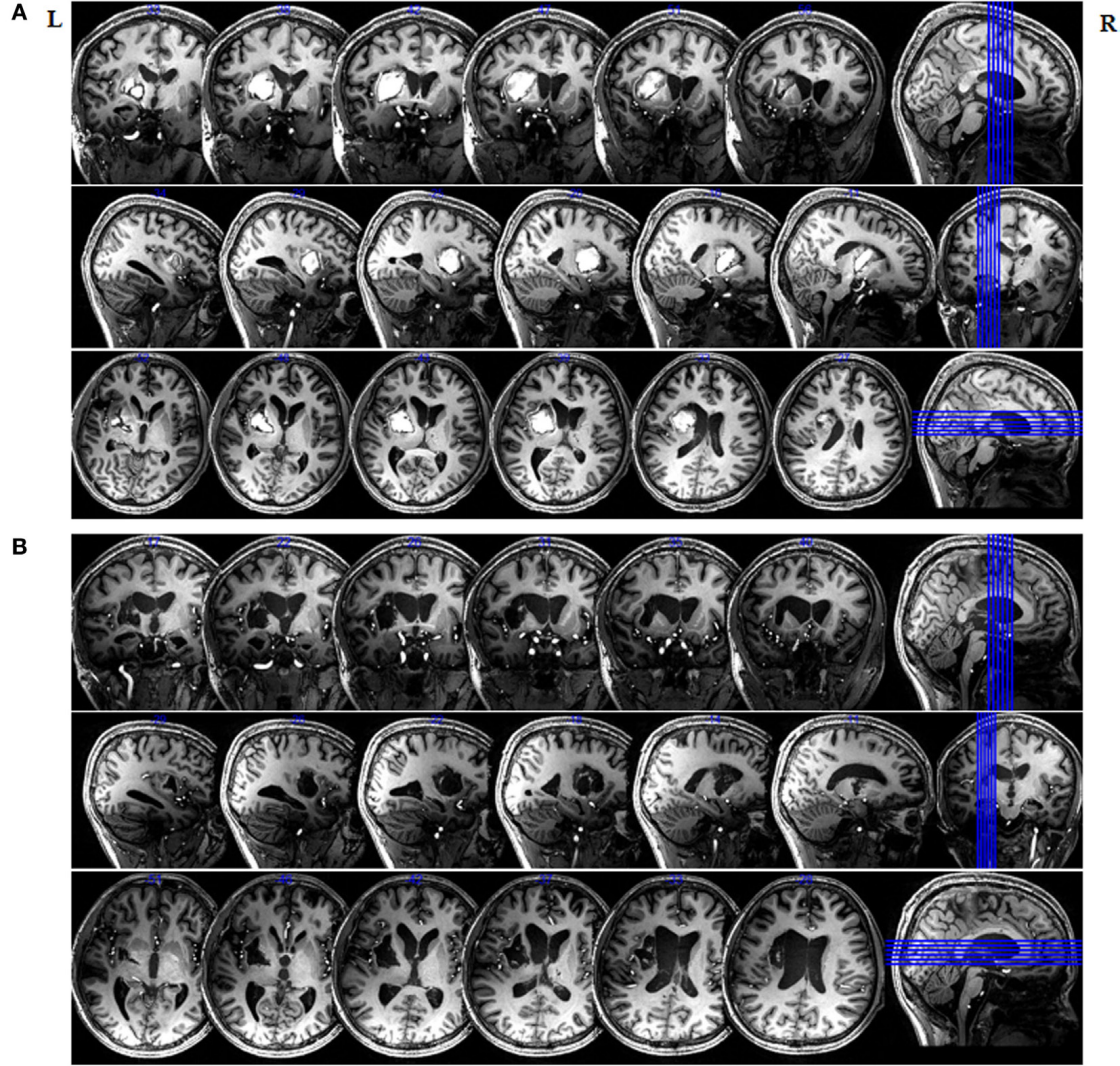
Magnetic resonance imaging (MRI) of the *in vivo* delineation of the patient’s entire brain at 7 T. 3D-T1-weighted sections of an MRI image of the entire brain obtained at **(A)** 1.5 months and **(B)** 5 months after initial injury.

Figure 1 clearly highlights the injured areas from their surroundings throughout the entire brain and indicates that the left hemisphere was mainly affected. Figure 1 also shows that the patient’s midline structure was slightly right shifted at one and a half months after onset, and an irregular high-T1WI signal is evident in the left basal ganglia and the left corona radiata. However, an irregular low-T1WI signal was also observed from the left basal ganglia and the left carona radiata on the second scan, which was taken 5 months after brain injury. Imaging showed that the left intracerebral hematoma region developed toward the malacia.

### Comparison of EEG Recordings between VS/UWS and MCS over the Long Term

In order to study changes in sleep architecture between the VS/UWS and MCS, we analyzed long-term EEG recordings obtained from the patient over multiple nights in both states. We focused on the spectral analysis of stage 2 sleep and spindling activity as described by Thengone et al. (4). At the first-time point of evaluation (VS/UWS), the sleep pattern of the patient was abnormal. The EEG was featured a diffuse slow wave in the theta frequency range and no obvious sleep spindling activity was observed. However, sleep spindling activities were clearly apparent on the evaluation taken during MCS (Figure S1 in Supplementary Material). Wave amplitude in the right temporal and right posterior occipital lobe increased significantly, along with smaller increases in the left temporal lobe and the left occipital follicle.

### Serial Comparison of White Matter between VS/UWS and MCS by Diffusion Connectometry

**Figure 2:**
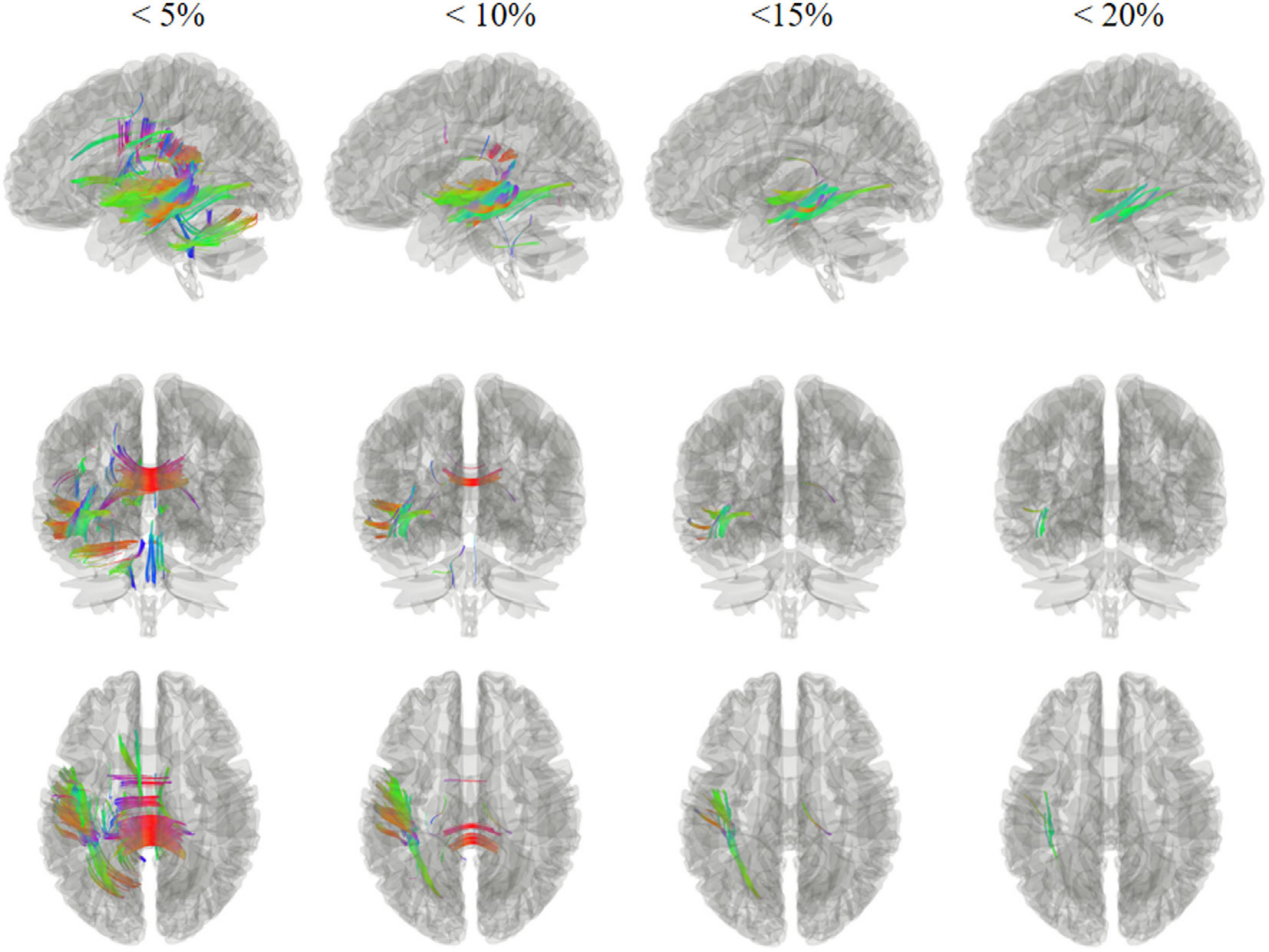
Tracts with significantly reduced density in the patient during minimally conscious state compared with vegetative state/unresponsive wakefulness syndrome. Specifically, the white matter regions exhibited a reduction mostly in the left hemisphere, and sub-regions of the corpus callosum. Red: left–right, green: anterior–posterior, blue: superior–inferior.

**Figure 3:**
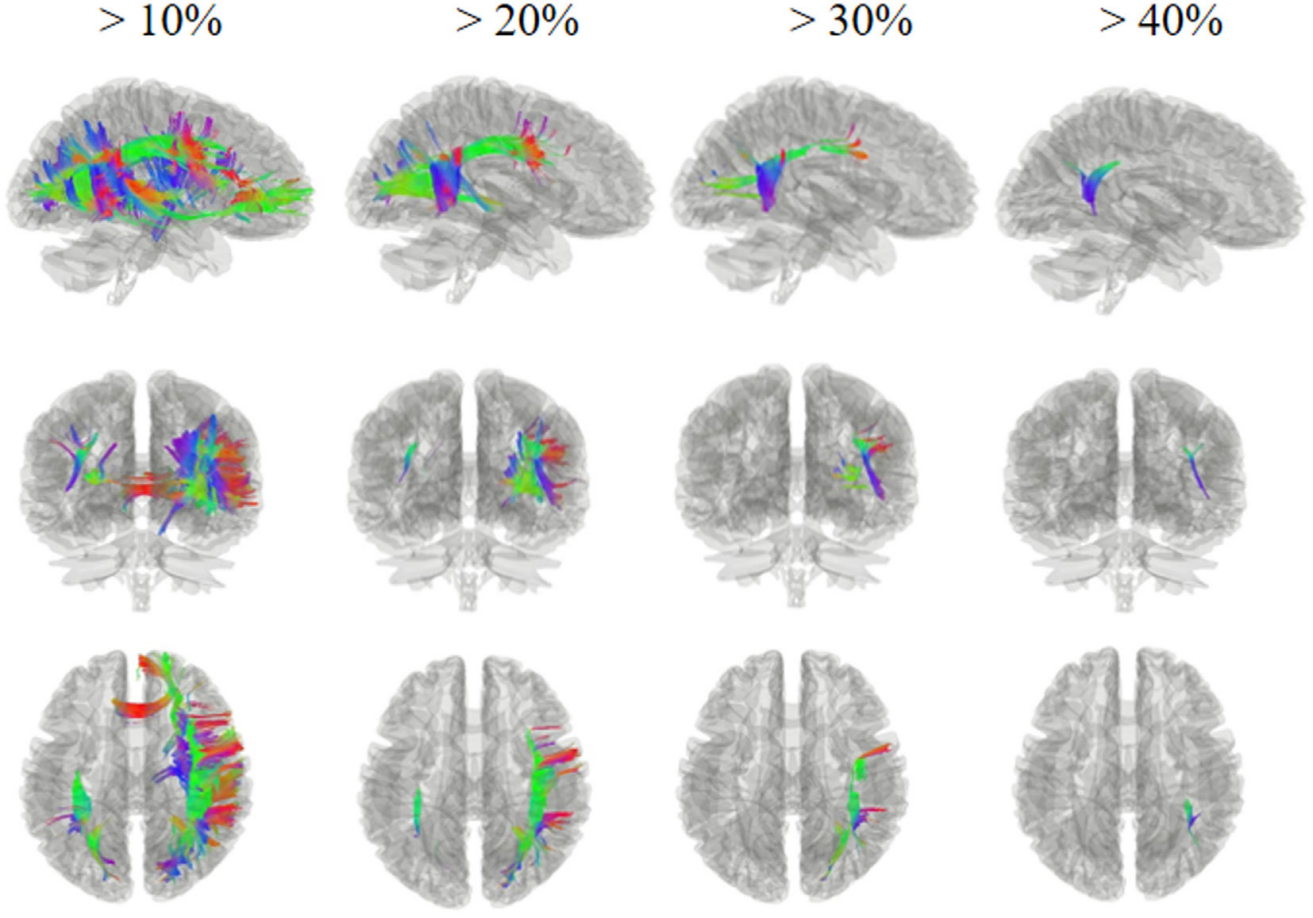
Tracts with significantly increased density in the patient during minimally conscious state compared with vegetative state/unresponsive wakefulness syndrome. The white matter regions exhibited an increase mostly in the right hemisphere, particularly the tracts of the right superior longitudinal fasciculus and the right arcuate fasciculus connecting the parietal, occipital, and temporal cortices; these showed a greater increase, of more than 30%, in the area of the temporoparietal junction. Red: left–right, green: anterior–posterior, blue: superior–inferior.

Figures 2 and 3 show the changes in connectometry results in addition to diffusion-weighted images from the patient in both states. Percentile ranking analysis was applied to the scans, which correspond to the VS/UWS and MCS, respectively. Ranked tract density was observed to decrease and increase during MCS when VS/UWS were plotted in sagittal, coronal, and axial views with directional color coding (red: left–right, green: anterior–posterior, blue: superior–inferior).

Tract density was reduced in the MCS when compared with the VS/UWS, and it was mostly evident in the left hemisphere, as demonstrated in Figure 2. Fibers showing reduced density included U fibers, the left inferior longitudinal fasciculus, the left middle longitudinal fasciculus, the left inferior fronto-occipital fasciculus, and the corpus callosum sub-regions. Most of these white matter regions exhibited more than a 5% reduction, with fibers in the left inferior longitudinal fasciculus and the left middle longitudinal fasciculus showing a reduction of more than 20%.

Figure 3 shows regions presenting proportional increases of 10, 20, 30, and 40%. In contrast to areas showing reduction, fibers with increased density were mostly located in the right hemisphere. These fibers included U fibers, the right arcuate fasciculus, the right cingulum, the right superior longitudinal fasciculus, and the corpus callosum sub-regions. In particular, the right superior longitudinal fasciculus and the right arcuate fasciculus, which connect the parietal, occipital, and temporal cortices, showed the largest increase with a greater than 30% increase in the area of the right temporoparietal junction (TPJ).

### Whole-Brain Distribution of Fibers with at least 10% Increases between VS/UWS and MCS

**Figure 4:**
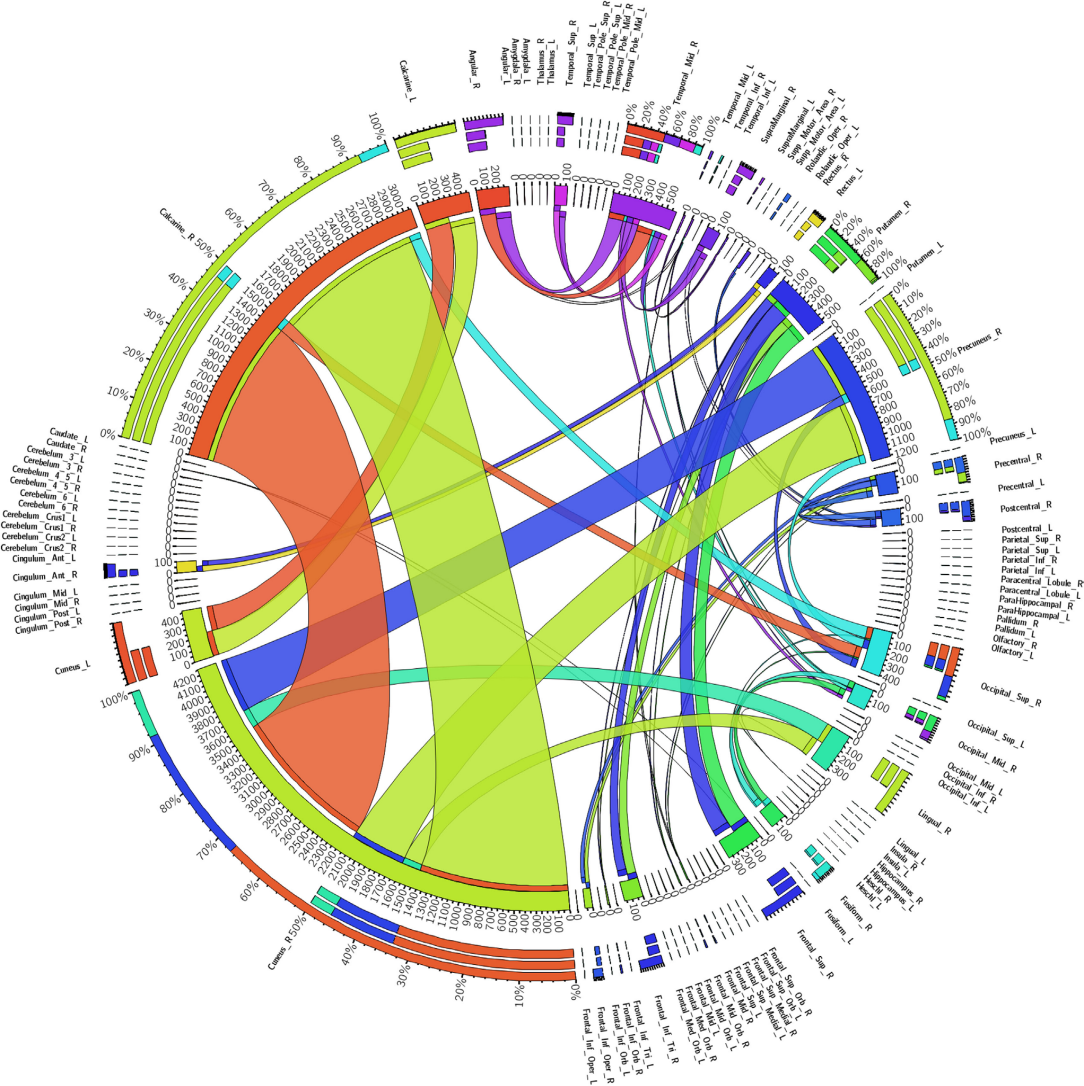
Regional weights and the distribution of 90 white matter connectivity sorted by the automated anatomical labeling-90. Regional weights and the degrees of connection are shown in the circular graph. Different numbers represent the exact fiber numbers or the proportions (as a percentage) of the fibers within one region. Ribbon size encodes the cell value associated with a column segment pair and ribbon ends are colored by column segment.

Figure 4 shows a fiber connectivity matrix in which the interconnection between the seeds is clearly demonstrated, along with the differences among different fibers. Several brain regions exhibited greater weights than others, for example, the cuneus_R, calcarine_R, precuneus_R, temporal_mid_R, and the putamen_R. The degree, which is defined for each seed as the number of links connected to this seed, was used to measure the importance of individual seeds (19). The temporal_mid_R exhibited the greatest degree (of 4), which implies that this region exhibited the highest increase of other seeds connected to it. The cuneus_R, occipital_sup_R, and the occipital_mid_R had a degree of 3, the second highest increase in the number of connections with other seeds.

## Discussion

Using connectometry analysis, this study characterized significant white matter changes occurring in a patient with DOC after improving from VS/UWS to MCS. First, in UWS/VS our patient preserved behavioral arousal (eyes could open and show a behavioral sleep–wake cycle), but self-awareness or awareness of the environment was absent, which meets the definition of UWS/VS (20, 21). However, the longitudinal EEG showed no obvious spindle wave. This is in line with the data previously reported by Landsness et al. (22), but differs from the study reported by Pavlov et al. (23) in which the majority of VS patients were found to retain some important circadian changes. The possible cause of this difference might be related to the severe brain damage in our patient. Later, sleep spindling activities were clearly apparent for this patient during MCS. An improvement of sleep spindling activities was evident from UWS/VS to MCS and was similar to the observations made by Fonseca et al. (24) in their follow-up of a patient with bilateral paramedian thalamic stroke. Such changes might be associated with the restoration of thalamocortical connectivity during the recovery of consciousness (25). We then determined how the properties of individual fibers changed when the patient moved from VS/UWS to MCS. Fibers exhibiting a reduced density were mostly distributed in the left hemisphere, where injury was the most severe. Fibers with an increased density were observed in both hemispheres, but were most prominent in the right hemisphere and were mostly associative fibers. More precisely, those with a greater proportional increase were mostly in the right TPJ, which contains the area encompassing the inferior parietal lobe, lateral occipital cortex, and posterior superior temporal sulcus (26). These results were in accordance with the active areas observed during 24-h EEG.

In whole-brain analyses, rather than individual fiber analysis, we compared fibers with at least a 10% proportional increase and observed that altered white matter connections with the highest discriminative weights were located within or across visual-related areas. Furthermore, the temporal_mid_R, which is associated with auditory sense, exhibited the highest increase of other seeds connected to it. These results suggest that fibers related to primary sensory areas underwent prominent increases in density during spontaneous recovery leading to an obvious improvement from VS/UWS to MCS, most particularly in vision-related areas, such as the cuneus and calcarine fissure and auditory-related areas, such as the temporal_mid_R. This was consistent with improvements to the visual and auditory components of the CRS-R which were greater than other improvements, and similar to the observations made in the case reported by Laureys et al. (27) which showed that FA increased in areas encompassing the cuneus and precuneus in a patient who remained in MCS for 19 years. These results are also consistent with a study reported by Thengone et al. (4) who described changes in a patient in local brain regions that supported language and visuomotor function over a period of 2 years and 9 months after severe brain injury. However, in our case, there was no obvious change in white matter related to language, and the CRS-R scores related to language varied; these results differed from those reported by Thengone et al. (4). As the main lesion was related to the left basal ganglia in this case, aphasia might be a secondary problem in this patient and deserves further investigation (28).

In our patient, the proportional increase in the right TPJ was the highest among the fibers compared serially between the VS/UWS and the MCS. The TPJ is implicated in a variety of processes including multisensory integration, social cognition, sense of agency, and stimulus-driven attention function (29) and has structural connections to many important areas (30–33). Although increases in density were observed in white matter in the left TPJ, the area and proportion of fibers affected were much lower than the right. The severity of injury in the left hemisphere may have been the main reason for this difference. Furthermore, the right and left TPJ are known to perform different functions (34). Thus, functional differences between hemispheres may also have contributed to this difference.

Furthermore, results in our case indicated that the highest weighted increases in MCS compared to VS were seen in the cuneus_R, followed by the calcarine_R. The temporal_mid_R exhibited the highest increase of other seeds connected to it, while the cuneus_R, occipital_sup_R, and occipital_mid_R all showed the second highest increase. The cuneus_R, calcarine_R, occipital_sup_R, and occipital_mid_R are subareas of the visual system. Recent studies have shown that visual fixation and visual pursuit are the commonest early clinical signs denoting MCS (35). Other researchers have confirmed that both coherent and incoherent movements significantly activate the cuneus and superior occipital gyrus (36), suggesting that these regions respond to wide-field visual motion. Density increases in the cuneus_R, occipital_sup_R, and the occipital_mid_R in our patient may have contributed to improvements observed in the perception of visual motion. Together, these results indicate that white matter density increases in the cuneus and calcarine might improve visual perception in patients with DOC. On the other hand, the middle temporal lobe, which is associated with the auditory cortex (37), plays a critical role in representing the motion information used for directional discrimination (38, 39). These findings may provide clues for studying the mechanisms of visual fixation and pursuit in DOC.

However, our study has two potential limitations. First, this was a single-subject study design and the patient’s left hemisphere was severely injured. Consequently, our results may not be applicable to trauma patients with a different pattern of injury. As a result, future studies should consider a diverse array of injuries across a greater number of patients. Second, our current study lacked event-related potentials (P300), which are particularly important in the study of DOC and might predict subsequent recovery of consciousness in unconscious patients (40, 41).

## Concluding Remarks

This was the first study to describe structural brain changes during spontaneous recovery from VS/UWS to MCS in a patient with severe trauma based on *in vivo* ultra-high field (7 T) MRI. Comparing identical fibers in MCS versus VS/UWS, we found that the number of fibers showing increased density, and the magnitude of change, were greatest in the area of the right TPJ. These results are in accordance with the active areas identified during 24-h EEG. Moreover, comparing different fibers exhibiting a 10% increase in density, or greater, across the entire brain, we found that altered white matter connections with the higher discriminative weights were located within or across visual-related areas, and that the temporal_mid_R, which is associated with the auditory cortex, exhibited the highest increase in connections to other seeds. This is consistent with the improvements noted on the CRS-R. Our findings provide a clear view of white matter dynamics during early recovery from VS/UWS to MCS, which may be informative for an early prognosis of recovery outcome and may help optimize treatment strategies of patients with DOC in the future.

## Ethics Statement

Our protocol was approved by the Ethics Committee of the First Affiliated Hospital, School of Medicine, Zhejiang University, and written informed consent was obtained from the legal guardian of the patient to allow the patient to participate in the study and for this case report to be published.

## Author Contributions

XT, RW, GP, and BL were responsible for the study design, literature search, and manuscript drafting. XT, JG, ZZ, and TG were responsible for data collection and statistical analysis. JL, FH, YW, and KL were mainly responsible for administrative, technical, or material support. XZ, GP, and BL were responsible for the study concept and critical revision. All authors contributed to discussions and reviewing of the manuscript.

## Conflict of Interest Statement

The authors declare that the research was conducted in the absence of any commercial or financial relationships that could be construed as a potential conflict of interest.
