# Supplementary material for: Spontaneous Recovery from Unresponsive Wakefulness Syndrome to a Minimally Conscious State: Early Structural Changes Revealed by 7-T Magnetic Resonance Imaging
Source: Front Neurol. 2018 Jan 17;8:741. doi: 10.3389/fneur.2017.00741 (PMC5776100; doi:10.3389/fneur.2017.00741)
Supplement: Supplementary file 1 [file image_1.PDF]

(A)

(B)

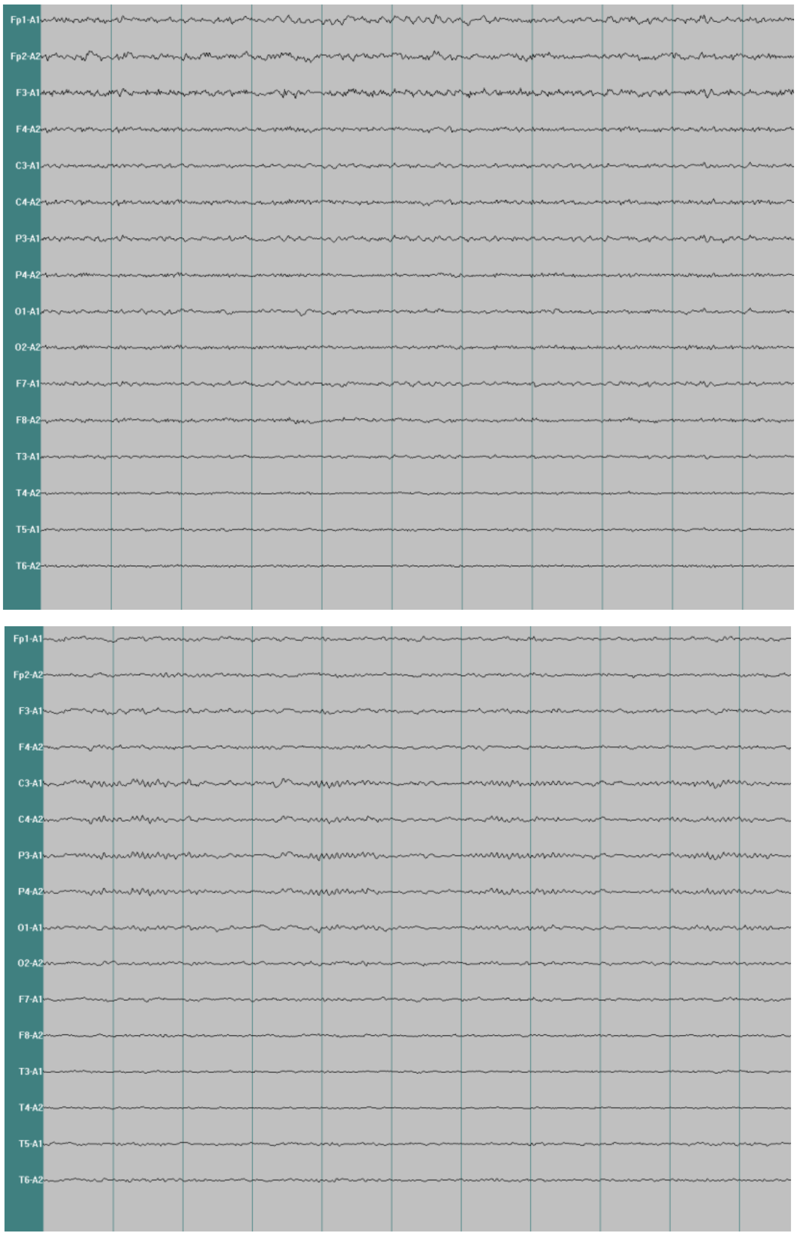

Figure S1 The comparing of the long-term electroencephalography (EEG) recordings at the VS state (A) and the MCS state (B). At the time point of (A) (VS state, 1.5 month after onset), EEG was composed of diffuse θ waves and no obvious sleep spindling activity was observed. However, at the evaluation of (B) (MCS state, 5 months after onset), obvious sleep spindling activities were observed clearly.
